# A Urinary Three-Metabolite Signature Enables Noninvasive Identification of Patients with High-Risk Ovarian Cancer

**DOI:** 10.1158/1078-0432.CCR-25-4260

**Published:** 2026-04-24

**Authors:** Alexander Max Funk, Mareike Brieske, Franziska Maria Schwarz, Theresa Link, Sophie Jonas, Pauline Wimberger, Lisa Freitag, Anna Klimova, Triantafyllos Chavakis, Peter Mirtschink, Jan Dominik Kuhlmann

**Affiliations:** 1 Institute for Clinical Chemistry and Laboratory Medicine, Faculty of Medicine and University Hospital Carl Gustav Carus, Technische Universität Dresden, Dresden, Germany.; 2 National Center for Tumor Diseases (NCT), NCT/UCC Dresden, a partnership between DKFZ, Faculty of Medicine and University Hospital Carl Gustav Carus, TUD Dresden University of Technology, and Helmholtz-Zentrum Dresden-Rossendorf (HZDR), Dresden, Germany.; 3 Department of Gynecology and Obstetrics, Faculty of Medicine and University Hospital Carl Gustav Carus, Technische Universität Dresden, Dresden, Germany.; 4 German Cancer Consortium (DKTK), Dresden and German Cancer Research Center (DKFZ), Heidelberg, Germany.

## Abstract

**Purpose:**

Reliable prognostic tools in ovarian cancer are urgently needed to guide risk-adapted treatment decisions, yet the clinical utility of urinary metabolites for noninvasive risk stratification remains largely undefined. Here, we define a clinically relevant urinary metabolite signature that enables noninvasive prognostic risk stratification in ovarian cancer.

**Experimental Design:**

We used targeted ^1^H nuclear magnetic resonance spectroscopy to profile 149 metabolites related to energy metabolism, oxidative stress, mitochondrial function, nitrogen metabolism, amino acid degradation, gut microbiome activity, and inflammation. Metabolites were measured in preoperative urine samples from 199 consecutive patients with newly diagnosed ovarian cancer treated in routine clinical practice between 2013 and 2022.

**Results:**

Unsupervised clustering revealed biologically heterogeneous subgroups but lacked prognostic resolution and alignment with overt clinical phenotypes. However, single-metabolite analysis identified a condensed three-metabolite prognostic signature comprising glycine, alanine, and citrate. A final parsimonious model integrating this metabolite signature with clinical covariates outperformed established risk factors alone (Fédération Internationale de Gynécologie et d'Obstétrique stage and surgical outcome), accurately predicted 60-month overall survival (AUC = 0.839), and stratified risk. Patients in the highest-risk quartile (Q4) had markedly shorter progression-free survival [Δ_median_ ≈ 56 months; HR, 2.63; 95% confidence interval (CI), 1.54–4.52; *P* < 0.001] and overall survival (Δ_median_ ≈ 86 months; HR, 2.49; 95% CI, 1.39–4.46; *P* = 0.009) compared with the lowest-risk group (Q1).

**Conclusions:**

We define a urinary three-metabolite signature that enables noninvasive identification of patients with high-risk ovarian cancer beyond established clinical factors. This signature may support molecular stratification and risk-adapted clinical decisions, thereby underscoring the clinical scalability of urine as a matrix for metabolic risk profiling in ovarian cancer.

**Translational Relevance**

Reliable prognostic biomarkers in ovarian cancer are urgently needed to guide risk-adapted treatment decisions. Although blood-based metabolomics has shown promise, urine represents a noninvasive, analytically accessible matrix that remains largely underexplored in the prognostic setting. In this study, we identify a urinary three-metabolite signature, comprising glycine, alanine, and citrate, that predicts overall survival independently of Fédération Internationale de Gynécologie et d'Obstétrique stage and surgical outcome. Glycine emerged as a robust marker of poor prognosis, whereas alanine and citrate were consistently associated with a favorable outcome. Notably, this urinary signature outperforms a previously established plasma-based model and offers nonredundant prognostic information. These findings highlight the biological distinctiveness and clinical potential of urinary metabolite profiling. Given its ease of collection and high patient acceptability, urinary metabolomics may be valuable for risk stratification in routine settings. Prospective validation of this marker panel may enable more personalized care in ovarian cancer, including earlier identification of high-risk patients who may benefit from intensified treatment.

## Introduction

Ovarian cancer remains the leading cause of mortality among women with gynecologic malignancies, with more than 70% of cases diagnosed at advanced stages (1). Improvements in surgical radicality and the clinical implementation of targeted therapies, including antiangiogenic agents such as bevacizumab and poly adenosine diphosphate-ribose polymerase (PARP) inhibitors, have significantly expanded treatment options for ovarian cancer (2–6). However, most patients still experience disease recurrence with poor long-term outcomes (3, 6). This persistent clinical challenge highlights the urgent need for novel noninvasive biomarkers that inform prognosis and stratify patients based on individual disease biology.

Metabolic reprogramming is a fundamental hallmark of malignant progression and plays a central role in ovarian cancer (7–9). In prior work, we performed blood-based metabolomics profiling to interrogate its clinical relevance in patients with ovarian cancer (10). Plasma profiling using targeted ^1^H nuclear magnetic resonance (NMR) identified two metabolite signatures, one reflecting lipid metabolism and the other associated with amino acid degradation, which were linked to differential patient outcomes. Further deconvolution markedly improved prognostic resolution and led to the identification of the acetoacetate^low^/3-hydroxybutyrate^low^/alanine^high^ metabolic profile as an independent predictor of superior clinical outcomes (10). Plasma metabolic signatures evolved dynamically during treatment, including normalization of key prognostic lipid metabolites. These changes point to underlying tumor-driven metabolic programs, likely reflecting both malignant activity and systemic host responses that imprint on systemic circulation and are dynamically reprogrammed by primary ovarian cancer treatment (10).

Although blood-based metabolomics captures clinically relevant systemic signals, urine offers distinct advantages: a less complex matrix, enrichment for low-molecular-weight metabolites, and greater sensitivity to dynamic metabolic alterations (11–13). Combined with its ease of collection, urine is ideally suited for routine clinical application. Despite growing interest in urinary metabolomics as a complementary tool for noninvasive biomarker discovery (14, 15), most studies in ovarian cancer have focused primarily on early detection (16), with limited evidence supporting its predictive and prognostic relevance. In addition, clinical translation of urinary metabolomics remains constrained by the scarcity of large, well-annotated patient cohorts, patient selection bias, methodologic inconsistencies, and variability across analytical platforms (17, 18). Furthermore, the degree of concordance between plasma- and urine-derived metabolic signatures remains poorly defined, and it is unclear whether tumor-associated metabolic alterations are reflected to a similar extent in both compartments, an essential consideration for developing integrated biomarker strategies (19).

In light of these limitations, we systematically assessed the predictive and prognostic relevance of urinary metabolites in ovarian cancer. To this end, we profiled urine samples from a clinically annotated patient cohort (*n* = 199) using a targeted panel of 149 metabolites implicated in energy metabolism, oxidative stress, mitochondrial dysfunction, nitrogen metabolism, amino acid degradation, gut microbiome activity, and inflammation. Additionally, we compared urinary and matched plasma-derived metabolic profiles to evaluate compartmental concordance and explore their combined utility for prognostic modeling.

## Materials and Methods

### Patient cohort and clinical characteristics

Patients were prospectively enrolled at the Department of Gynecology and Obstetrics, Technische Universität Dresden, Germany. A total of 199 consecutive patients with histologically confirmed primary epithelial ovarian, peritoneal, or fallopian tube carcinoma diagnosed between 2013 and 2022 were included (Table 1). All patients underwent cytoreductive surgery with the goal of achieving macroscopic complete tumor resection, followed by platinum-based adjuvant chemotherapy in accordance with national treatment guidelines. Enrollment in clinical trials was permitted. Median follow-up time, overall survival (OS), and progression-free survival (PFS) were calculated from the date of primary diagnosis. The study was approved by the Institutional Review Board of Technische Universität Dresden (reference number EK74032013) and conducted in accordance with the Declaration of Helsinki, Good Clinical Practice guidelines, and applicable national regulations. Written informed consent was obtained from all participants. Tumor classification was performed according to the World Health Organization criteria for female genital tract tumors, and disease staging was based on the revised 2014 Fédération Internationale de Gynécologie et d'Obstétrique (FIGO) system. FIGO staging was applied retrospectively to cases diagnosed before 2014. Patients with advanced-stage disease (FIGO III–IV) were eligible for antiangiogenic therapy with bevacizumab, administered concomitantly with chemotherapy and continued as maintenance therapy. Surgical outcomes were classified as either macroscopic complete resection or residual disease. For comparison, urine samples from a smaller cohort of healthy women (*n* = 19) were collected and processed using the same workflow and normalization procedure described below.

**Table 1.** Patient characteristics at primary diagnosis.

Characteristic	Category	Value
Patients	​	199
Age (years)	Median (range)	63 (23–84)
FIGO stage (%)	I/II	54 (27.1)
​	III/IV	145 (72.9)
Tumor type (%)	HGSOC	125 (62.8)
​	LGSOC	7 (3.5)
​	Other tumor types	67 (33.7)
Surgical debulking (%)	Residual disease	63 (31.7)
​	No residual disease	133 (66.8)
Histology (%)	Serous	143 (71.9)
​	Nonserous	44 (22.1)
BMI (%)	≥25	106 (53.3)
​	<25	86 (43.2)
BRCA 1/2 mutational status (%)	BRCA1/2wt	54 (27.1)
​	BRCA1/2mut	37 (18.6)
​	Unknown	108 (54.3)
Follow-up time	Months [median (95% CI)]	81.7 (74.4–95)
PFS	Months [median (range)]	34.5 (0.5–205.1)
​	Progression/death (%)	120 (60.3)
​	No progression/death (%)	79 (39.7)
OS	Months [median (range)]	51.6 (0.5–205.1)
​	Dead (%)	100 (50.3)
​	Alive (%)	99 (49.7)
CA-125 (U/mL)	Preoperative concentration [median (range)]	365 (6.5–14207)
Bevacizumab (%)	Bevacizumab maintenance treatment	94 (47.2)
​	No bevacizumab maintenance treatment	105 (52.8)
PARPi (%)	PARPi treatment (documented)	34 (17.1)
​	No documented PARPi (includes untreated or blinded allocation)	165 (82.9)

Abbreviations: BMI, body mass index; CA-125, cancer antigen 125; HGSOC, high-grade serous ovarian cancer; LGSOC, low-grade serous ovarian cancer; mut, mutant; PARPi, PARP inhibitor; wt, wild-type.

### Plasma sampling and preparation

Detailed plasma sample processing, metabolite detection, and data preprocessing procedures are described in the recently published companion study (10). In the present study, these published plasma data were used exclusively for cross-compartment comparison; all urine metabolomics measurements and all statistical analyses were performed independently as described below.

### Urine sampling and preparation

Preoperative urine samples were collected in 10-mL S-Monovette tubes (Sarstedt AG & Co.). All included patients had evaluable preoperative urine profiling by targeted ^1^H NMR and were therefore assessable for the urine assay (199/199; 100%). Samples were centrifuged at 1,800 × *g* for 8 minutes at room temperature. The supernatant was immediately aliquoted and stored at −80°C until further analysis, minimizing freeze–thaw cycles. For metabolomics profiling, samples were thawed on ice and processed immediately upon complete thawing. Laboratory staff were blinded to all clinical information during sample handling.

### Targeted urine metabolomics profiling

Targeted NMR spectroscopy was performed following established protocols (20). Urine samples were mixed (540 µL) with phosphate buffer containing trimethylsilylpropanoic acid (60 µL) as an internal standard, yielding a final volume of 600 µL, and transferred into NMR tubes. Spectra were acquired on a Bruker Avance III Neo 600-MHz spectrometer equipped with a BBI probe and a SampleJet autosampler with 4°C storage capacity. Measurements were conducted at 300 K according to Bruker’s in vitro diagnostics research (IVDr) standard operating procedures. Data acquisition and processing were performed automatically using TopSpin 4.1.1 (RRID: SCR_014227) and ICON-NMR software. Metabolite quantification was conducted using Bruker IVDr B.I.Quant-UR E (v1.1.0). The analyzed metabolites, including their assigned functional classes, along with corresponding limits of detection (LOD), are summarized in Supplementary Table S1.

### Statistical analysis

All statistical analyses were performed using MetaboAnalyst 6.0 (RRID: SCR_015539; ref. 21) and R v4.4.2 (R Core Team; ref. 22). Data were retrieved from an SQL database using the DBI and RSQLite R packages for subsequent analysis. Visualizations were created using MetaboAnalyst 6.0 (21) and R v4.4.2 (R Core Team; ref. 22). To attenuate dilution-related variability in urinary samples, all values were normalized to creatinine concentration (23). Metabolites with normalized signals exceeding the LOD in ≥ 50% of samples were retained. Before statistical analysis, data were preprocessed differently. For the analysis in MetaboAnalyst, missing values were replaced by the feature’s mean. In R, a complete-case approach was used, in which entire samples containing missing metabolite values were excluded. Metabolite data for MetaboAnalyst were log10-transformed and autoscaled, whereas for R analysis, the data were only autoscaled. Unsupervised principal component analysis and K-means clustering were used to explore global variation in metabolite profiles. Sparse partial least squares discriminant analysis was applied to identify cluster-defining metabolites based on variable loadings. Associations between individual metabolites or metabolic signatures and clinical parameters were evaluated using Mann–Whitney U test, χ^2^ test of Independence, and Fisher exact test, as appropriate. Kaplan–Meier survival curves were generated and compared using the (pairwise) log-rank test. The median follow-up time was calculated by reverse Kaplan–Meier analysis. We used Cox-LASSO, a penalized regression method, for simultaneous variable selection and mode regularization. The multivariable Cox proportional hazards regression models were used to assess associations with OS and PFS, adjusting for established risk factors like FIGO stage and surgical outcome. We performed the likelihood ratio test (LRT) to compare nested Cox models. Additionally, a prognostic risk score was calculated for each patient from the model’s linear predictor: Risk score = $\sum_{i=1}^{p}\beta_{i}X_{i}$, in which $\beta_{i}$ are the Cox model coefficients and $X_{i}$ are the metabolite values (24). For survival analysis, patients were subsequently stratified into quartiles (Q1–Q4) based on the risk score distribution. For cross-compartment urine–plasma concordance analyses, we included only metabolites detected above the LOD in ≥ 50% of plasma samples and quantified in both matrices. No additional urine LOD threshold was applied for this comparison to preserve biologically informative urine–plasma discordance, including low or undetected urinary values. To account for different concentration ranges and units across biofluids, metabolite concentrations were z-standardized within each compartment, and Pearson correlation coefficients were calculated on the matched z-standardized values. Time-dependent ROC analysis was performed to evaluate model discriminative performance for 60-month OS. To account for censoring before 60 months, area-under-the-curve (AUC) estimates were calculated using inverse probability of censoring weighting with marginal Kaplan–Meier estimation of the censoring distribution, as implemented in the R package “timeROC” (version 0.4; RRID: SCR_026444). Hazard ratios (HR) and 95% confidence intervals (CI) were reported. Exact procedures will be available upon request. The primary packages used for data processing, statistical analysis, and visualizations were as follows: ggplot2 (v. 3.5.2; RRID: SCR_014601), ggpubr (v. 0.6.0; RRID: SCR_021139), survminer (v. 0.5.0; RRID: SCR_021094), survival (v. 3.8.3; RRID: SCR_021137), glmnet (v. 4.1.8; RRID: SCR_015505), pheatmap (v. 1.0.13; RRID: SCR_016418), corrplot (v. 0.95; RRID: SCR_024683), dplyr (v. 1.1.4; RRID: SCR_016708), timeROC (v. 0.4; RRID: SCR_026444), car (v. 3.1.3; RRID: SCR_022137), carData (v. 3.0.5), rms (v. 8.0.0; RRID: SCR_023242), dbi (v. 1.2.3), and RSQLite (v. 2.3.11; refs. 25–30).

## Results

### The urinary metabolome encodes prognostic information at single-metabolite resolution but not at the cluster level

**Figure 1.**
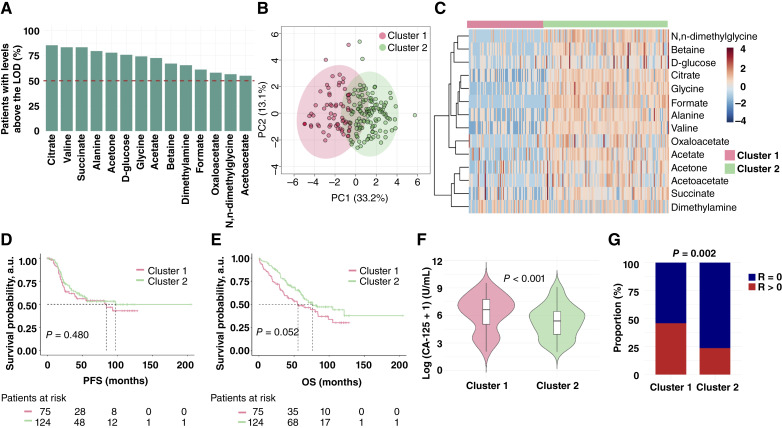
Urine-based metabolomics profiling in patients with ovarian cancer at the cluster level. **A,** Metabolites with ≥ 50% nonzero values across urine samples from 199 patients with ovarian cancer. **B,** Principal component (PC) analysis of urinary metabolites, identifying two distinct metabolic clusters (cluster 1: red; cluster 2: green). **C,** Mean metabolite abundances across clusters. **D,** PFS and (**E**) OS by metabolic cluster assignment. **F,** Cancer antigen 125 (CA-125) levels and (**G**) surgical outcome [macroscopic complete resection (*R* = 0) vs. residual disease (*R* > 0)] stratified by cluster. Statistical significance was assessed by Wilcoxon or χ^2^ test, as appropriate; exact *P* values are shown in the panels.

We performed targeted ^1^H NMR–based metabolomics profiling of urine samples collected at the time of primary diagnosis from patients with ovarian cancer (*n* = 199). The deidentified metabolite-level dataset underlying the reported analyses is provided in Supplementary Data S1. To ensure data quality, the initial set of 149 metabolites was filtered for analytical robustness. A metabolite was retained for subsequent analysis only if its normalized signal exceeded the LOD in ≥ 50% of samples (Supplementary Fig. S1; Supplementary Table S1). This filtering step yielded a final set of 14 metabolites, representing a focused subset of the measurable urinary metabolome in this cohort (Fig. 1A). These include intermediates of central energy metabolism (e.g., citrate, succinate, D-glucose, acetate, and oxaloacetate), amino acids or amino acid-related compounds (e.g., valine, alanine, and glycine), and metabolites involved in one-carbon and methylation pathways (e.g., betaine, dimethylglycine, formate, and dimethylamine). In addition, ketone body-related metabolites (acetone and acetoacetate) are represented, likely capturing diverse aspects of tumor-associated metabolic reprogramming at diagnosis. Among these, citrate, valine, and succinate were the most consistently detected across the cohort, suggesting a prominent contribution to the urinary metabolome in patients with ovarian cancer (Fig. 1A).

To investigate interpatient variability in urinary metabolomics profiles, we performed unsupervised k-means clustering based on the quantified metabolites. This analysis stratified the cohort into two groups, cluster 1 (*n* = 75) and cluster 2 (*n* = 124; Fig. 1B and C), each defined by a distinct urinary metabolic signature. The clustering was independent of potential confounding variables, including urine storage duration, patient age, and body mass index (Supplementary Fig. S2A–S2C). Notably, clinicopathologic characteristics such as FIGO stage and breast cancer gene 1/2 (BRCA1/2) mutational status were also evenly distributed between the two clusters (Supplementary Fig. S2D and S2E). Although a trend toward prognostic separation was observed, cluster assignment was not statistically associated with clinical outcome [PFS: HR, 0.85 (95% CI, 0.54–1.33), *P* = 0.479; OS: HR, 0.68 (95% CI, 0.46–1.01), *P* = 0.052], indicating that the observed metabolic profiles do not reflect overt clinical phenotypes (Fig. 1D and E). Nevertheless, the clusters correlated with serum cancer antigen 125 levels and showed an association with surgical outcome (*P* < 0.001; *P* = 0.002; Fig. 1F and G), suggesting a weak preoperative association with subsequent debulking efficiency. Whether this clustering pattern also tracks postoperative residual tumor burden cannot be evaluated in the absence of postoperative urine sampling.

**Figure 2.**
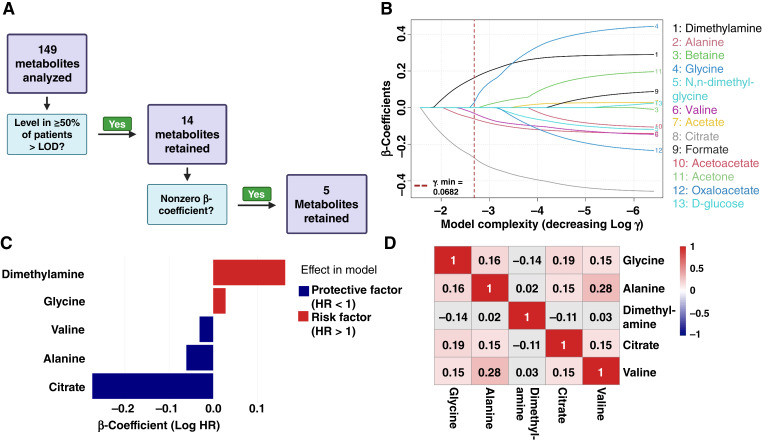
Identification of prognostically informative urinary metabolites. **A,** Overview of the analytic workflow used to identify urinary metabolites with prognostic relevance. **B,** Regularization path of Cox-LASSO regression across λ-values. **C,** Nonzero β-coefficients at optimal λ (λ_min) for retained metabolites. Here, β denotes the Cox regression model coefficient and is shown as the log HR for each metabolite, not the metabolite concentration. **D,** Pairwise Pearson correlations among retained metabolites (*n* = 190); all *r* < 0.28. (**A,** Image created with BioRender.com. Schwarz, F. [2026] https://BioRender.com/7oq101y.)

Given the absence of a consistent prognostic signal at the cluster level, we next examined associations between individual urinary metabolites and OS. To identify the most informative predictors, we applied penalized Cox regression to the full panel of 14 metabolites retaining five metabolites, namely glycine, citrate, dimethylamine, alanine, and valine (Fig. 2A–C). These metabolites exhibited minimal intercorrelation (all pairwise *r* < 0.28; Fig. 2D), supporting their inclusion in composite models. Among the five retained metabolites, higher concentrations of citrate [Cox model β coefficient (log HR) = −0.274], alanine [Cox model β coefficient (log HR) = −0.061], and valine [Cox model β coefficient (log HR) = −0.031] were associated with improved OS. In contrast, elevated levels of dimethylamine [Cox model β coefficient (log HR) = 0.163] and glycine [Cox model β coefficient (log HR) = 0.028] correlated with reduced OS (Fig. 2C). Functionally, the five retained metabolites span key biochemical domains, including amino acid metabolism (glycine, alanine, and valine), mitochondrial and energy pathways (citrate), and nitrogen/methyl group handling (dimethylamine), suggesting that diverse metabolic processes contribute to interindividual variation in clinical outcome.

Taken together, these findings suggest that urinary metabolic clustering reflects biologically heterogeneous subgroups but lacks prognostic resolution and alignment with overt clinical phenotypes. In contrast, individual metabolite-level concentration analysis identified five urinary markers of clinical outcome.

### Prognostic modeling identifies a three-metabolite urinary signature that improves risk stratification beyond clinical parameters

**Figure 3.**
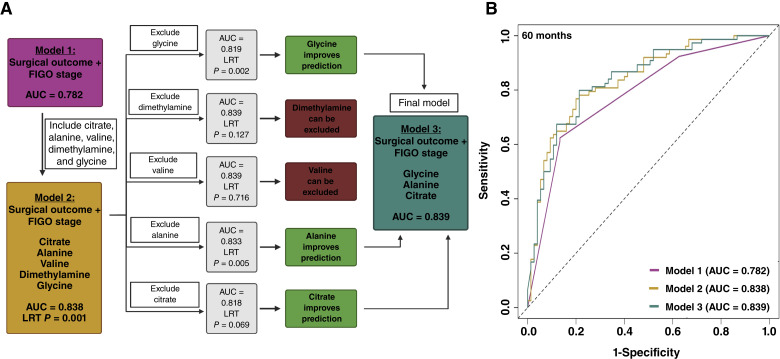
Identification of an optimized three-metabolite signature. **A,** Representation of stepwise model construction, comparing nested Cox models and ROC performance. **B,** Time-dependent ROC curves of the clinical model (model 1), extended model (model 2: clinical +5 metabolites), and optimized model (model 3: clinical + glycine, alanine, citrate). AUC values indicate discriminatory performance for 60-month OS. To account for individuals censored before 60 months, AUC estimates were calculated using the inverse probability of censoring weighting method. Analysis was restricted to the complete-case cohort. (**A,** Image created with BioRender.com. Schwarz, F. [2026] https://BioRender.com/jd4n0h0.)

To derive a parsimonious yet prognostically informative urinary metabolite signature, we applied a stepwise model reduction strategy starting from our five preselected candidates (glycine, alanine, citrate, dimethylamine, and valine; Figs. 2 and 3; Supplementary Table S2). Therefore, we focused the analysis on 60-month OS, a clinically established endpoint for long-term outcome prediction and risk stratification in ovarian cancer. As an initial benchmark, we constructed a clinical reference model (Fig. 3; model 1) comprising surgical outcome and FIGO stage as the most relevant prognostic parameters in advanced ovarian cancer. This baseline model achieved an AUC of 0.782. We next developed an extended model (Fig. 3; model 2) by incorporating clinical covariates and all five urinary metabolites. The inclusion of metabolic features significantly enhanced predictive performance, raising the AUC from 0.782 to 0.838, thereby outperforming the clinical reference model and providing added prognostic value. This highlights the complementary prognostic contribution of urinary metabolites beyond established clinical risk factors. To further refine the model and to eliminate redundancy, we conducted a systematic backward elimination from model 2, evaluating the relevance of each included metabolite through likelihood ratio testing and changes in AUC relative to the full model (Fig. 3; Supplementary Fig. S3; Supplementary Table S2). The removal of dimethylamine and valine did not affect the model’s performance (*P* > 0.1), indicating a lack of independent prognostic value. In contrast, exclusion of glycine and alanine led to a significant decline in the model’s accuracy (*P* ≤ 0.005), confirming their essential role in the signature. Citrate provided a consistent incremental contribution across two nested model comparisons (LRT *P* = 0.068–0.069) and was therefore retained as the third component of the parsimonious signature. This process resulted in a final prognostic model (model 3) that included the three most informative metabolites: glycine, alanine, and citrate alongside the clinical parameters surgical outcome and FIGO stage. This refined model maintained high predictive accuracy with an AUC of 0.839, while using fewer variables, creating a more efficient and clinically practical signature with improved interpretability (Fig. 3).

**Figure 4.**
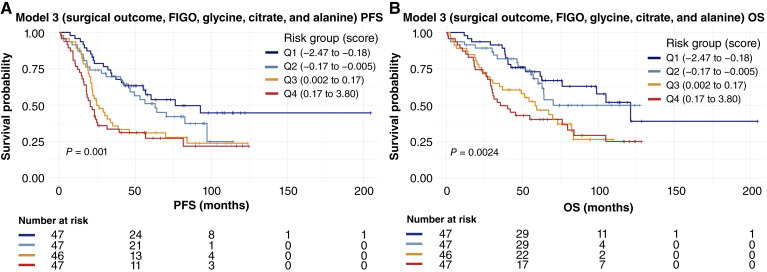
Prognostic stratification using the three-metabolite signature in combination with clinical covariates (model 3). Kaplan–Meier curves for (**A**) PFS and (**B**) OS across quartiles of the composite risk score derived from model 3. The log-rank test was used to assess statistical significance.

To illustrate its translational utility, we fitted the final model 3 (comprising glycine, alanine, citrate, FIGO stage, and surgical outcome), as a multivariable Cox proportional hazards model and derived a continuous risk score from its linear predictor (see Experimental Design). Metabolite concentrations were z-standardized, and higher scores corresponded to increased risk. Within this model, urinary glycine remained a statistically independent adverse prognostic marker (HR, 1.42; 95% CI, 1.21–1.67; *P* < 0.0001), whereas alanine (HR, 0.79; 95% CI, 0.61–1.03; *P* = 0.08) and citrate (HR, 0.79; 95% CI, 0.61–1.02; *P* = 0.072) did not meet conventional significance thresholds but contributed to overall model performance and were therefore retained. Patients were stratified into quartiles based on the risk score (Q1–Q4) for Kaplan–Meier and log-rank analyses (Supplementary Table S3 for OS distribution within quartiles; Table S4 for risk score range and quartile cutoffs). This revealed marked survival differences between groups, with a Δ_median_ of ∼56 months for PFS (Q4 vs. Q1: HR, 2.63; 95% CI, 1.54–4.52; *P* < 0.001; Fig. 4A) and ∼86 months for OS (Q4 vs. Q1: HR, 2.49; 95% CI, 1.39–4.46; *P* = 0.009; Fig. 4B), confirming the stratification capacity of the three-metabolite signature when integrated with standard clinical parameters.

To address potential confounding by maintenance therapy and histologic subtype, we extended the prognostic analyses by annotating bevacizumab and PARP inhibitor exposure and assessing both metabolite-level interactions and the prognostic performance of the derived risk score across treatment and histologic subgroups. The score remained prognostic in patients treated with bevacizumab and in those not treated with bevacizumab, with no evidence of interaction (*P* interaction = 0.68). Likewise, there was no significant interaction with PARP inhibitor exposure (*P* interaction = 0.407), noting limited power in the clearly exposed PARP subgroup due to frequent trial-related blinding. The score also remained prognostic in both high-grade serous ovarian cancer (HGSOC) and non-HGSOC tumors, with no evidence of effect modification by histology (*P* interaction = 0.424; Supplementary Tables S5–S7). To further assess whether the prognostic effect of the score itself was independent of clinicopathologic and treatment-related variables not included in score construction, we fitted additional multivariable validation models using the derived risk score, entered either as a continuous or median-dichotomized variable and adjusted for histology, bevacizumab exposure, and PARP inhibitor exposure, which confirmed an independent association with OS (continuous HR, 3.13; 95% CI, 1.95–5.03; *P* < 0.001; dichotomized HR, 1.91; 95% CI, 1.23–2.97; *P* = 0.004). Together, these analyses support robustness of the urinary signature across contemporary maintenance strategies and major histologic subtypes.

As further biological context, creatinine-normalized urinary concentrations of alanine, citrate, and glycine did not differ significantly between a healthy female control cohort (*n* = 19) and patients with ovarian cancer (*n* = 199; alanine *P* = 0.086; citrate *P* = 0.36; glycine *P* = 0.089; Wilcoxon rank-sum test; Supplementary Fig. S4), supporting that the prognostic signal is unlikely to reflect a strong case–control shift and is more consistent with interpatient variability within ovarian cancer.

In summary, we identified a robust urinary signature of glycine, alanine, and citrate that enhances risk stratification beyond established clinical factors in ovarian cancer.

### The three-metabolite urinary signature predicts response to platinum-based chemotherapy in an exploratory post hoc analysis

To explore whether the urinary signature relates to chemotherapy response, we compared individual metabolic risk scores between patients with platinum-resistant relapse and those without such early relapse. Platinum-resistant relapse was defined as relapse within 6 months after completion of first-line platinum-based chemotherapy and was operationalized in this cohort as PFS from primary diagnosis < 12 months. Patients meeting this platinum-resistant definition exhibited significantly higher metabolic risk scores (Supplementary Fig. S5).

These data suggest that the urinary signature may also have predictive potential for platinum-based chemotherapy response; however, this represents a post hoc exploratory analysis.

### Urinary metabolome outperforms plasma signature in prognostic modeling

To assess compartment-specific concordance between the urinary and plasma metabolomes, we compared urinary metabolite concentrations with previously profiled matching plasma samples from the same patients using the targeted ^1^H NMR platform and preprocessing workflow described in our companion plasma study (10). The cohort included 161 patients for whom both urine and plasma samples were available. To enable cross-compartment concordance analysis, we selected metabolites that were detected above the LOD in ≥ 50% of plasma samples and were also quantified in both urine and plasma. No detection threshold was applied to urine, allowing the identification of discordant profiles. This approach reflects the asymmetric application of LOD thresholds to plasma and urine, ensuring systemic relevance while preserving biologically meaningful nondetections in urine, and yielded 19 metabolites for comparative analysis.

**Figure 5.**
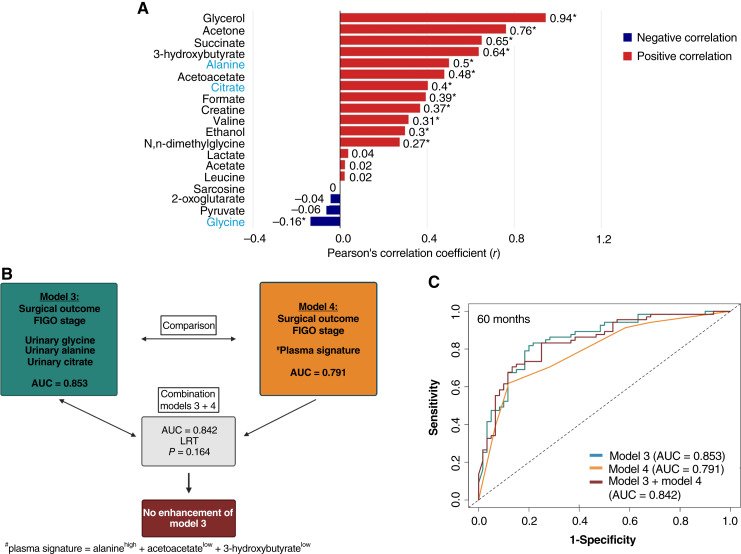
Comparative analysis of urinary versus plasma-based metabolomic signature. **A,** Pairwise correlations between metabolite concentrations in matched plasma and urine samples (*n* = 161); r denotes Pearson’s coefficient. Asterisks indicate *P* < 0.05. **B,** Schematic of model comparisons using urinary metabolites and a previously established plasma signature. **C,** Time-dependent ROC curves of the urinary model, plasma signature, and combined model. AUC values indicate discriminatory performance for 60-month OS. To account for censoring before 60 months, AUC estimates were calculated using inverse probability of censoring weighting. Analysis was restricted to the complete-case cohort. (**B,** Image created with BioRender.com. Schwarz, F. [2026] https://BioRender.com/dy8x8k4.)

To enable direct comparability across differing units and concentration ranges, all metabolite concentrations were z-standardized. For the majority of metabolites, concentrations in urine were broadly aligned with those in plasma, indicating that a substantial fraction of the plasma metabolome is mirrored in the urine. Notably, glycerol, acetone, and succinate exhibited strong and statistically significant correlations (*r* > 0.6), consistent with systemic regulation and efficient renal clearance. However, a smaller subset of metabolites showed only weak or inconsistent correlations (*r* < 0.3; Fig. 5A). For example, pyruvate and acetate displayed no significant concordance between urine and plasma concentrations. We next focused on glycine, alanine, and citrate, the three metabolites constituting our final urinary prognostic signature, and assessed the relationship between their urinary and plasma concentrations. Urinary alanine and citrate showed a moderate positive correlation with their corresponding concentrations in plasma (*r* = 0.50, *r* = 0.40, respectively), indicating partial systemic reflection. In contrast, the concentration of glycine in urine showed a weak negative correlation with that in plasma (*r* = −0.16), suggesting compartment-specific regulation or differential renal clearance (Fig. 5A).

To assess whether urinary and plasma metabolomics signatures convey overlapping or complementary prognostic information, we compared nested Cox regression models incorporating clinical covariates (FIGO stage and surgical outcome), our newly derived urinary signature (urinary glycine, alanine, and citrate; Fig. 3), and a previously established and optimized plasma signature [plasma acetoacetate^low^, 3-hydroxybutyrate^low^, and alanine^high^ (10)]. Direct comparison revealed superior prognostic performance of the urinary model over the plasma model (AUC = 0.853 vs. 0.791). We then tested whether combining both biofluid-based signatures would yield additional prognostic value. However, inclusion of the plasma signature into the urinary model did not significantly improve its prognostic performance (Δχ^2^ = 1.93, *P* = 0.164; Fig. 5B and C; Supplementary Table S8). These findings support at least the nonsuperiority of the combined model and suggest that the prognostic information conveyed by the plasma signature is largely redundant to that already captured by the urinary profile.

Collectively, these analyses demonstrate that, despite partial overlap between the urinary and plasma metabolomes, their prognostic contributions are distinct. The urine-based signature outperforms its plasma counterpart in stratifying patient risk, highlighting its superior prognostic resolution in ovarian cancer. These results suggest that, for metabolomics risk stratification, urine alone may be sufficient.

## Discussion

We systematically profiled urinary metabolites to assess their prognostic relevance in ovarian cancer. The finding that individual metabolites, rather than broader metabolic clusters, conveyed prognostic information suggests biologically specific, compartment-level influences. This aligns with prior studies reporting that clinically informative signals often reside in single analytes or small subsets of two to three metabolites rather than in large panels. In colorectal cancer, for instance, a two-metabolite signature (diacetylspermine and kynurenine) matched the performance of a 17-metabolite model (31), whereas in renal cell carcinoma, N-formylkynurenine alone approximated the accuracy of a nine-metabolite panel (32). Similar patterns have emerged from transcriptomic analyses, in which models based on individual genes frequently achieve predictive power comparable with that of multigene classifiers (33). These findings suggest that discrete metabolite-level perturbations may more precisely capture tumor–host interactions than broader pathway-level patterns and underscore the utility of prioritizing individual metabolite concentrations in biomarker discovery, particularly for urinary profiling in ovarian cancer.

Previous urine-based studies in ovarian cancer have primarily focused on early detection and diagnostic discrimination (16). Prognostic urinary biomarkers for ovarian cancer remain scarce, with previously reported candidates such as neopterin (34) reflecting immune activation rather than core metabolites, which yield more distinct and quantifiable signals in NMR spectra. Although TCA cycle intermediates are enriched in the urine of patients with ovarian cancer (35), their prognostic value remains unestablished. Our study is the first to demonstrate that a preoperative urine-based signature comprising glycine, alanine, and citrate predicts survival independently of clinical risk factors and outperforms a matched plasma-based metabolomics model. All ^1^H NMR measurements were conducted under certified laboratory-developed protocols, ensuring analytic reproducibility and scalability for clinical application. Our findings establish urine as a biologically proximate, noninvasively accessible, and clinically informative matrix for capturing metabolic features that are prognostically relevant yet orthogonal to established clinical risk factors. By focusing on individual metabolite concentrations, our approach enables molecular risk stratification based on a compact, interpretable model. The superior prognostic performance of this three-metabolite panel compared with plasma-based models underscores the clinical relevance of urinary profiling. From a translational perspective, our signature may support early identification of high-risk patients and inform risk-adapted decisions such as treatment intensification, closer surveillance, or trial prioritization within the contemporary therapeutic framework of ovarian cancer (36). However, our study is prognostic and does not justify selection of PARP inhibitors or anti-VEGF therapy based on the metabolite signature. Moreover, our findings are hypothesis-generating, and prospective validation in independent cohorts, ideally within clinical trial settings, is required before pursuing clinical implementation. Key limitations include the retrospective, single-center design, which is susceptible to selection bias, treatment-era effects, and residual confounding that cannot be fully eliminated despite multivariable adjustment and sensitivity analyses. In addition, the exploratory analysis relating the risk score to platinum-resistant relapse may be affected by overfitting and optimism bias and will require prospective confirmation using predefined response endpoints. Overall, prospective validation and integration into routine oncology practice are critical next steps, and future validation studies of this prognostic signature should adhere to REMARK reporting principles for tumor marker prognostic studies (37).

Although this study was not designed to resolve causality, the consistent and statistically robust association of glycine, alanine, and citrate with clinical outcomes suggests the involvement of underlying pathophysiologic processes. The prognostic associations observed for these urinary metabolites likely reflect systemic metabolic stress linked to tumor biology and host response. Aligning with the high one-carbon and nucleotide demand of proliferating tumors (38, 39), increased systemic glycine turnover raises filtered delivery, which may exceed the reabsorptive capacity of proximal tubular transporters such as SLC6A18 (40), thereby reducing fractional reabsorption and leading to urinary accumulation. Conversely, reduced urinary alanine may reflect increased tumor uptake and systemic utilization, exemplified by SLC38A2-mediated import in pancreatic cancer (41), which lowers the filtered load while proximal reabsorption remains unsaturated, resulting in urinary alanine depletion. Similarly, decreased urinary citrate plausibly reflects acid–base sensitive renal handling and preferential metabolic retention under high energetic and biosynthetic demand, with tumoral utilization of extracellular citrate supporting growth (42, 43). Collectively, although increased tumor uptake or turnover may help explain the observed urinary patterns, these signals may also reflect a broader physiologic context involving host, stromal, and hepatic metabolism, all of which influence renal handling (44, 45). Thus, urinary levels of glycine, alanine, and citrate may serve as a systemic readout of tumor- and host-mediated metabolic reprogramming associated with adverse clinical outcome. Notably, unlike canonical oncometabolites such as 2-hydroxyglutarate (46), which result from tumor-specific mutations and act locally, these metabolites are not tumor-specific and participate in fundamental metabolic processes. This may enhance prognostic utility by capturing both tumor-driven and host-mediated alterations, representing a key strength of our signature.

We observed substantial variability in urine–plasma correlations across metabolites, reflecting differences in physicochemical properties and renal handling. Glycerol showed the highest urine–plasma concordance, consistent with free glomerular filtration of a small, unbound solute and proportional proximal tubular reabsorption via the aquaglyceroporin AQP7, consistent with the preserved linearity we observed between compartments in this range (47–49). In contrast, glycine showed a weak inverse correlation, despite free filtration, consistent with regulated, sodium-dependent reabsorption via proximal tubular transporters such as SLC6A18 (40). Variable fractional reabsorption in response to filtered load or transporter dynamics may thereby decouple urinary excretion from plasma levels. These contrasting examples underscore that renal handling critically shapes urine–plasma relationships and must be carefully considered when interpreting compartment-specific metabolite profiles for biomarker discovery.

Moreover, we found that the prognostic relevance of metabolites differs between compartments, as exemplified by glycine, which is prognostic in urine but not in plasma. This likely reflects compartment-specific accumulation shaped by anatomic proximity and differential clearance. Ovarian cancer spreads within the peritoneal cavity, placing tumors near the urinary tract (50). Although tumor-derived metabolites may enter the bloodstream, degradation, uptake, or protein binding can limit their plasma detectability. In contrast, urine may accumulate these compounds over time, enhancing analytic accessibility. A similar concept has been described in renal cell carcinoma, in which tumor proximity facilitates urinary detection of metabolic alterations (51). Elevated glycine flux, linked to tumor proliferation (39), may therefore manifest more readily in urine than in plasma, potentially accounting for its exclusive prognostic relevance in the urinary compartment. Alanine showed prognostic relevance in both compartments (10), consistent with its conserved role in cancer metabolism. In line with these findings, the superior performance of the urine-based model underscores the metabolic distinctness of biofluids and supports compartment-specific biomarker strategies in ovarian cancer.

In conclusion, we identify a clinically relevant three-metabolite urinary signature in patients with ovarian cancer that improves prognostic stratification beyond established clinical risk factors. Our findings position urine as a scalable, biologically distinct matrix for noninvasive metabolic profiling with potential implications for treatment intensification, tailored surveillance, and integration of targeted therapies.

## Supplementary Material

Supplementary Figures Supplementary Figures 1-5.

Supplementary Tables Supplementary Tables 1-8.

Supplementary Data 1 Urine Metabolite Dataset.

## Data Availability

The deidentified processed metabolite-level data underlying the findings of this study are provided in Supplementary Data S1. This file contains the quantified urinary metabolite measurements for all analyzed samples together with the deidentified sample annotations required to reproduce the reported analyses. Because the dataset is compact and can be shared in full with the article, it has been provided directly as supplementary data rather than through a separate external repository. The individual-level clinical outcome data are not publicly available because public release would compromise patient privacy. Deidentified data may be available from the corresponding author upon reasonable request, subject to ethical and data protection requirements.
