# Supplementary material for: A Urinary Three-Metabolite Signature Enables Noninvasive Identification of Patients with High-Risk Ovarian Cancer
Source: Clin Cancer Res. 2026 Apr 24;32(14):2968–78. doi: 10.1158/1078-0432.CCR-25-4260 (PMC13376881; doi:10.1158/1078-0432.CCR-25-4260)
Supplement: Supplementary Figures — 1-5. [file ccr-25-4260_supplementary_figures_suppsf.pptx]

## Slide 1
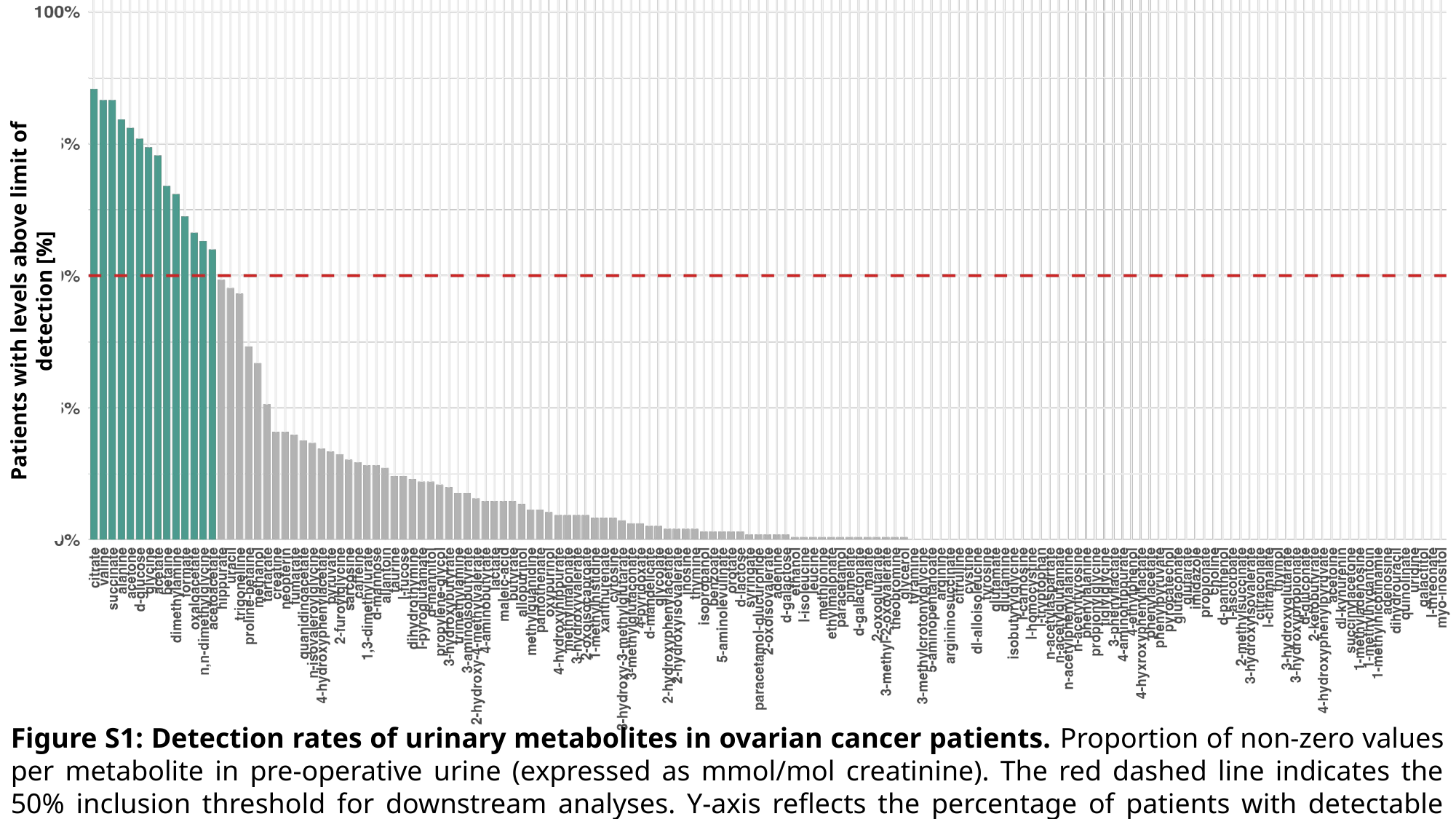

Patients with levels above limit of detection [%]
Figure S1: Detection rates of urinary metabolites in ovarian cancer patients. Proportion of non-zero values per metabolite in pre-operative urine (expressed as mmol/mol creatinine). The red dashed line indicates the 50% inclusion threshold for downstream analyses. Y-axis reflects the percentage of patients with detectable concentrations.

## Slide 2
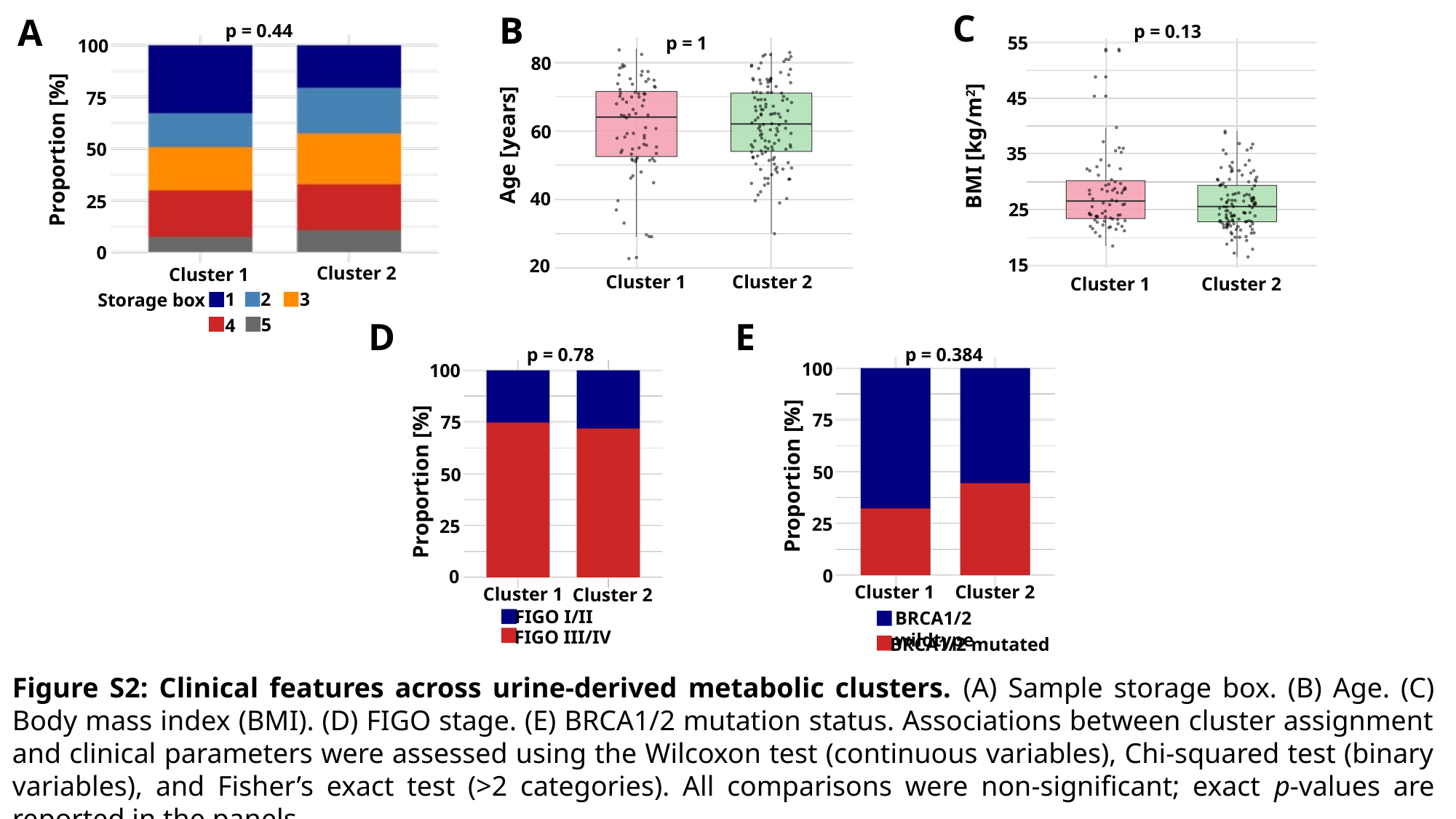

C
B
A
p = 0.44
p = 0.13
p = 1
55
45
BMI [kg/m2]
35
25
15
Cluster 2
Cluster 1
100
75
Proportion [%]
50
25
0
Cluster 2
Cluster 1
1
2
3
5
4
Storage box
80
60
Age [years]
40
20
Cluster 2
Cluster 1
D
E
p = 0.78
p = 0.384
100
75
50
Proportion [%]
25
0
Cluster 1
Cluster 2
BRCA1/2 wildtype
BRCA1/2 mutated
100
75
50
Proportion [%]
25
0
Cluster 1
Cluster 2
FIGO I/II
FIGO III/IV
Figure S2: Clinical features across urine-derived metabolic clusters. (A) Sample storage box. (B) Age. (C) Body mass index (BMI). (D) FIGO stage. (E) BRCA1/2 mutation status. Associations between cluster assignment and clinical parameters were assessed using the Wilcoxon test (continuous variables), Chi-squared test (binary variables), and Fisher’s exact test (>2 categories). All comparisons were non-significant; exact p-values are reported in the panels.

## Slide 3
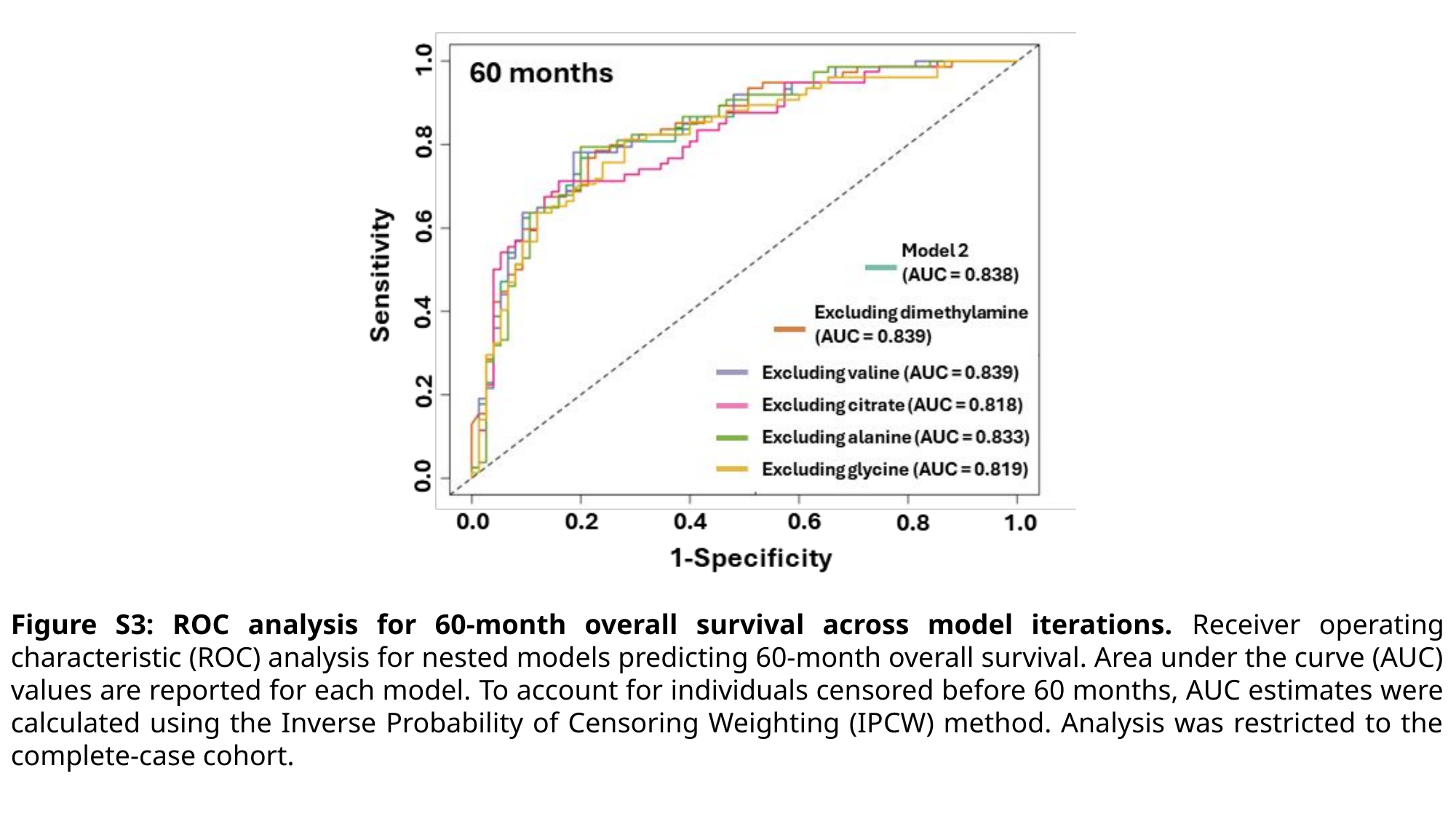

Figure S3: ROC analysis for 60-month overall survival across model iterations. Receiver operating characteristic (ROC) analysis for nested models predicting 60-month overall survival. Area under the curve (AUC) values are reported for each model. To account for individuals censored before 60 months, AUC estimates were calculated using the Inverse Probability of Censoring Weighting (IPCW) method. Analysis was restricted to the complete-case cohort.

## Slide 4
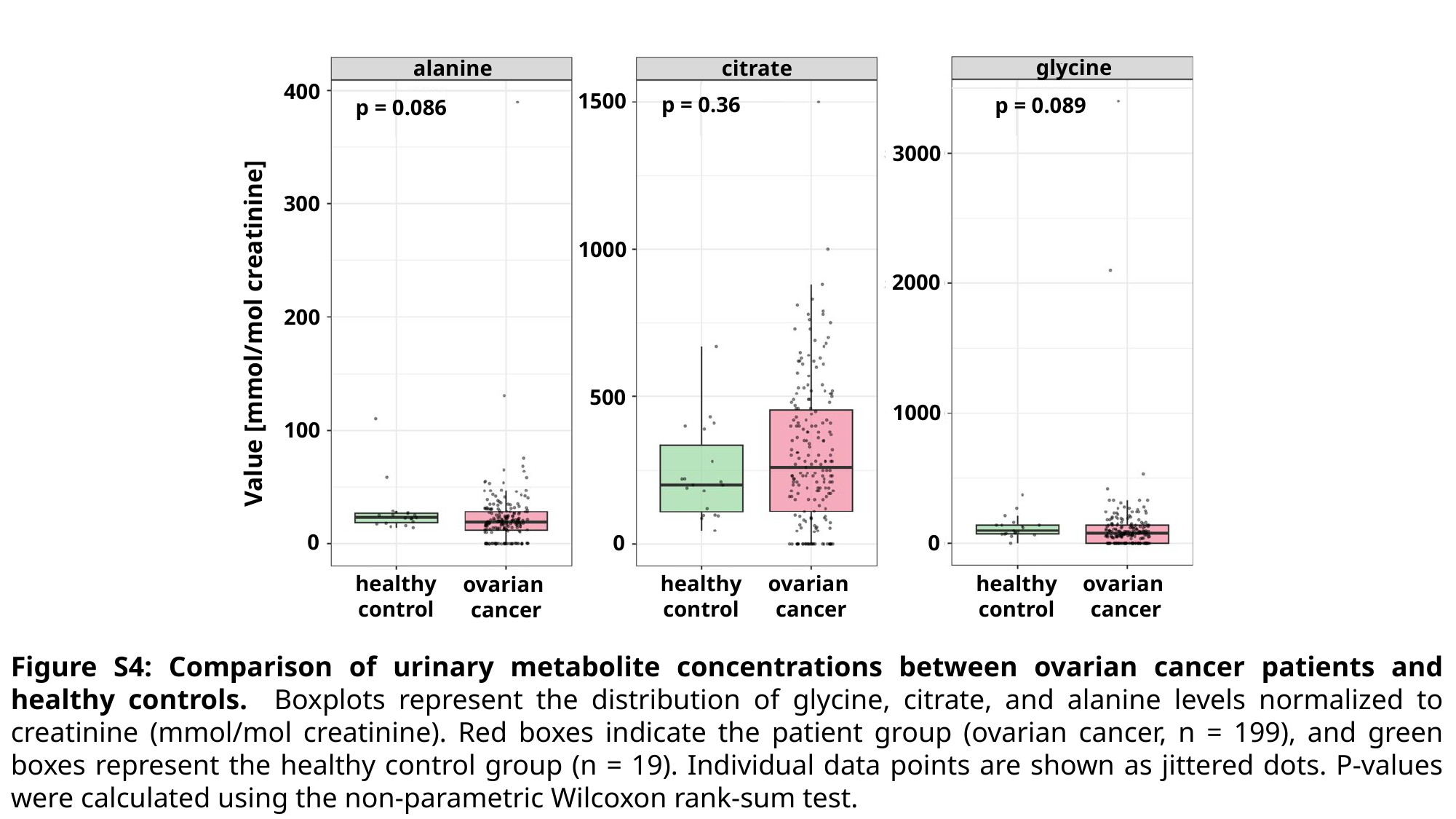

glycine
p = 0.089
3000
2000
1000
0
healthy
control
ovarian
cancer
alanine
400
p = 0.086
300
200
Value [mmol/mol creatinine]
100
0
healthy
control
ovarian
cancer
citrate
p = 0.36
500
0
healthy
control
ovarian
cancer
1500
1000
Figure S4: Comparison of urinary metabolite concentrations between ovarian cancer patients and healthy controls. Boxplots represent the distribution of glycine, citrate, and alanine levels normalized to creatinine (mmol/mol creatinine). Red boxes indicate the patient group (ovarian cancer, n = 199), and green boxes represent the healthy control group (n = 19). Individual data points are shown as jittered dots. P-values were calculated using the non-parametric Wilcoxon rank-sum test.

## Slide 5
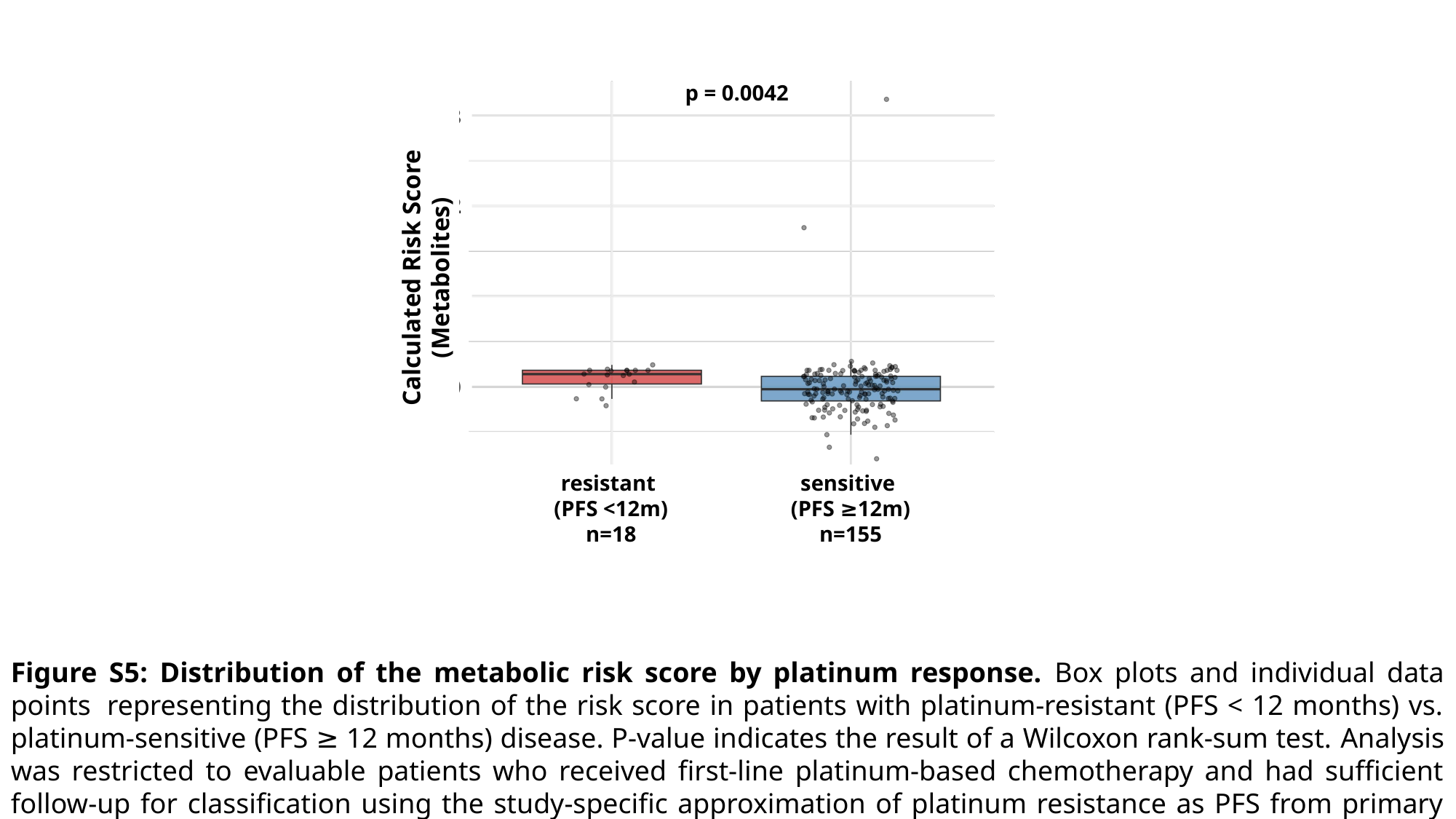

p = 0.0042
3
2
Calculated Risk Score (Metabolites)
1
0
resistant
(PFS <12m)
n=18
sensitive
(PFS ≥12m)
n=155
Figure S5: Distribution of the metabolic risk score by platinum response. Box plots and individual data points  representing the distribution of the risk score in patients with platinum-resistant (PFS < 12 months) vs. platinum-sensitive (PFS ≥ 12 months) disease. P-value indicates the result of a Wilcoxon rank-sum test. Analysis was restricted to evaluable patients who received first-line platinum-based chemotherapy and had sufficient follow-up for classification using the study-specific approximation of platinum resistance as PFS from primary diagnosis < 12 months (n=173).
