# Supplementary material for: A Urinary Three-Metabolite Signature Enables Noninvasive Identification of Patients with High-Risk Ovarian Cancer
Source: Clin Cancer Res. 2026 Apr 24;32(14):2968–78. doi: 10.1158/1078-0432.CCR-25-4260 (PMC13376881; doi:10.1158/1078-0432.CCR-25-4260)
Supplement: Supplementary Tables — 1-8. [file ccr-25-4260_supplementary_tables_suppst.docx]

**Table S1: Limit of detection (LOD) of the analyzed metabolites in the urine.**

| ***Category*** | ***Compound*** | ***LOD (mmol/mol creatinine)*** |
| --- | --- | --- |
| alcohols and derivatives | ethanol | 70 |
|  | isopropanol | 3 |
|  | methanol | 48 |
|  | propylene glycol | 39 |
| amines and derivatives | 1-methylguanidine | 64 |
|  | dimethylamine | 31 |
|  | trimethylamine | 2 |
|  | tyramine | 80 |
| amino acids and derivatives | 1-methylhistidine | 15 |
|  | 2-furoylglycine | 39 |
|  | 3-aminoisobutyric acid | 85 |
|  | 3-methylcrotonylglycine | 8 |
|  | 4-aminobutyric acid | 20 |
|  | 5-aminopentanoic acid | 94 |
|  | alanine | 10 |
|  | arginine | 750 |
|  | argininosuccinic acid | 29 |
|  | betaine | 7 |
|  | citrulline | 690 |
|  | creatine | 50 |
|  | cystine | 490 |
|  | dl-alloisoleucine | 48 |
|  | dl-tyrosine | 44 |
|  | glutamic acid | 460 |
|  | glutamine | 440 |
|  | glycine | 34 |
|  | guanidinoacetic acid | 100 |
|  | isobutyrylglycine | 7 |
|  | l-carnosine | 130 |
|  | l-homocystine | 910 |
|  | l-isoleucine | 16 |
|  | l-pyroglutamic acid | 32 |
|  | l-tryptophan | 97 |
|  | leucine | 22 |
|  | methionine | 18 |
|  | n,n-dimethylglycine | 5 |
|  | n-acetylaspartic acid | 99 |
|  | n-acetylglutamate | 42 |
|  | n-acetylphenylalanine | 130 |
|  | n-acetyltyrosine | 380 |
|  | n-isovaleroylglycine | 2 |
|  | phenylalanine | 200 |
|  | proline betaine | 25 |
|  | propionylglycine | 12 |
|  | sarcosine | 2 |
|  | taurine | 140 |
|  | tiglylglycine | 19 |
|  | valine | 2 |
| benzene and substituted derivatives | 2-hydroxyphenylacetic acid | 10 |
|  | 3-phenyllactic acid | 89 |
|  | 4-aminohippuric acid | 270 |
|  | 4-ethylphenol | 13 |
|  | 4-hydroxyhippuric acid | 26 |
|  | 4-hydroxyphenylacetic acid | 18 |
|  | 4-hydroxyphenyllactic acid | 43 |
|  | benzoic acid | 10 |
|  | d-mandelic acid | 2 |
|  | hippuric acid | 170 |
|  | phenylacetic acid | 72 |
|  | phenylpyruvic acid | 98 |
|  | pyrocatechol | 170 |
|  | syringic acid | 38 |
| carboxylic acids | 5-aminolevulinic acid | 2 |
|  | acetic acid | 5 |
|  | citric acid | 40 |
|  | e-glutaconic acid | 38 |
|  | ethylmalonic acid | 35 |
|  | formic acid | 10 |
|  | fumaric acid | 2 |
|  | glutaric acid | 170 |
|  | imidazole | 48 |
|  | lactic acid | 49 |
|  | maleic acid | 4 |
|  | methylmalonic acid | 17 |
|  | propionic acid | 51 |
|  | succinic acid | 5 |
|  | tartaric acid | 5 |
|  | trigonelline | 35 |
|  | xanthurenic acid | 18 |
| cosmetics, vitamines, drugs and drug metabolites | choline | 46 |
|  | d-panthenol | 17 |
|  | l-ascorbic acid | 160 |
|  | pantothenic acid | 16 |
|  | paracetamol | 50 |
|  | paracetamol-glucuronide | 24 |
| fatty acids and derivatives | 2-hydroxy-4-methylvaleric acid | 7 |
|  | 2-hydroxyisovaleric acid | 4 |
|  | 2-methylsuccinic acid | 48 |
|  | 3-hydroxy-3-methylglutaric acid | 19 |
|  | 3-hydroxyisovaleric acid | 36 |
|  | 3-hydroxyvaleric acid | 2 |
|  | 3-methylglutaconic acid | 16 |
|  | butyric acid | 15 |
|  | citraconic acid | 37 |
|  | l-citramalic acid | 100 |
|  | pimelic acid | 31 |
|  | thymol | 44 |
| hydroxy acids and derivatives | 3-hydroxyglutaric acid | 49 |
|  | 3-hydroxypropionic acid | 35 |
|  | d-galactonic acid | 57 |
|  | d-gluconic acid | 99 |
|  | glycolic acid | 180 |
|  | malic acid | 97 |
| keto acids and derivatives | 2-ketobutyric acid | 68 |
|  | 2-oxoglutaric acid | 92 |
|  | 2-oxoisocaproic acid | 5 |
|  | 2-oxoisovaleric acid | 9 |
|  | 3-hydroxybutyric acid | 100 |
|  | 3-methyl-2-oxovaleric acid | 29 |
|  | 4-hydroxyphenylpyruvic acid | 50 |
|  | acetoacetic acid. | 14 |
|  | acetoine | 14 |
|  | acetone | 2 |
|  | dl-kynurenin | 790 |
|  | oxaloacetic acid | 17 |
|  | pyruvic acid | 9 |
|  | succinylacetone | 130 |
| purine, pyridine and pyrimidine derivatives | 1,3-dimethyluric acid | 17 |
|  | 1-methyladenosine | 5 |
|  | 1-methylhydantoin | 42 |
|  | 1-methylnicotinamide | 32 |
|  | 4-pyridoxic acid | 11 |
|  | adenine | 10 |
|  | adenosine | 390 |
|  | allantoin | 17 |
|  | allopurinol | 10 |
|  | caffeine | 45 |
|  | cytosine | 5 |
|  | dihydrothymine | 94 |
|  | dihydrouracil | 710 |
|  | inosine | 19 |
|  | neopterin | 23 |
|  | orotic acid | 5 |
|  | oxypurinol | 20 |
|  | quinolinic acid | 68 |
|  | theobromine | 120 |
|  | thymine | 5 |
|  | uracil | 50 |
|  | uridine | 19 |
| sugars and derivatives | d-galactose | 43 |
|  | d-glucose | 34 |
|  | d-lactose | 96 |
|  | d-mannitol | 180 |
|  | d-mannose | 6 |
|  | galactitol | 370 |
|  | glycerol | 190 |
|  | l-fucose | 300 |
|  | l-threonic acid | 320 |
|  | myo-inositol | 4400 |

**Table S2: Likelihood-ratio test (LRT) of nested cox regression for different models.**

| ***comparison*** | ***Δdf*** | ***Δχ²*** | ***p.value*** |
| --- | --- | --- | --- |
| M1: surgical outcome + FIGO vs  M2: surgical outcome + FIGO + citrate + alanine + valine +  dimethylamine + glycine | 5 | 20.24 | 0.0011** |
| M2 vs M2 without glycine | 1 | 9.69 | 0.0019** |
| M2 vs M2 without dimethylamine | 1 | 2.33 | 0.127 |
| M2 vs M2 without valine | 1 | 0.13 | 0.716 |
| M2 vs M2 without alanine | 1 | 8.04 | 0.0046* |
| M2 vs M2 without citrate | 1 | 3.31 | 0.069 |
| M3: surgical outcome+ FIGO + glycine + alanine + citrate vs M3 without citrate | 1 | 3.33 | 0.068 |
| M3 vs M3 without alanine | 1 | 7.32 | 0.0068** |
| M3 vs M3 without glycine | 1 | 9.26 | 0.0023** |

*All metabolites are z standardized. Δχ² = difference in the chi-square statistic between two nested models (equivalent to 2 × Δ log-likelihood).*

**Table S3: Overall survival according to metabolite risk quartile.**

| ***Risk group*** | ***Median OS (95%CI)*** |
| --- | --- |
| Q1 | 121.26 (76.25-NA) |
| Q2 | 70.37 (63.04-NA) |
| Q3 | 58.51 (37.03-83.6) |
| Q4 | 35.61 (29.90-84.07) |

OS = overall survival, CI = confidence interval.

**Table S4: Distribution of the metabolite risk score across quartiles.**

| ***Risk group*** | ***n*** | ***Median risk score*** | ***Lower 25th quartile*** | ***Upper 75th quartile*** | ***Minimal risk score*** | ***Maximal risk score*** |
| --- | --- | --- | --- | --- | --- | --- |
| Q1 | 47 | -0.33 | -0.45 | -0.24 | -2.47 | -0.18 |
| Q2 | 47 | -0.08 | -0.12 | -0.04 | -0.17 | -0.005 |
| Q3 | 46 | 0.08 | 0.02 | 0.14 | 0.002 | 0.17 |
| Q4 | 47 | 0.31 | 0.23 | 0.32 | 0.17 | 3.80 |

**Table S5A: Univariate Cox analysis of bevacizumab for overall survival.**

| **HR (95% CI)** | ***p.value*** |
| --- | --- |
| 1.638 (1.066-2.519) | 0.025 |

*HR = hazard ratio, CI = confidence interval.*

**Table S5B: Multivariable Cox model including the three-metabolite signature, clinical risk factors, and bevacizumab.**

|  | **HR (95% CI)** | ***p.value*** |
| --- | --- | --- |
| **Surgical outcome** | 4.133 (2.473-6.907) | < 0.001 |
| **FIGO** | 2.55 (1.175-5.533) | 0.018 |
| **Bevacizumab** | 0.825 (0.514-1.325) | 0.427 |
| **Glycine** | 1.451 (1.227-1.716) | < 0.001 |
| **Citrate** | 0.821 (0.623-1.082) | 0.161 |
| **Alanine** | 0.749 (0.541-1.037) | 0.081 |

*Metabolite concentrations were z-standardized; Hazard Ratios (HR) refer to an increase of one standard deviation. Reference categories: No tumor residual (NoRest), FIGO I/II, no bevacizumab. CI = confidence interval.*

**Table S6A: Univariate Cox analysis of PARP inhibitor therapy for overall survival.**

| **HR (95% CI)** | **p.value** |
| --- | --- |
| 1.34 (0.73-2.43) | 0.345 |

*HR = hazard ratio, CI = confidence interval.*

**Table S6B: Multivariable Cox model including the three-metabolite signature, clinical risk factors, and PARP inhibitor therapy.**

|  | **HR (95% CI)** | ***p.value*** |
| --- | --- | --- |
| **Surgical outcome** | 4.06 (2.44-6.75) | < 0.001 |
| **FIGO** | 2.32 (1.10-4.88) | 0.027 |
| **PARP inhibitor** | 0.99 (0.54-1.81) | 0.968 |
| **Glycine** | 1.45 (1.23-1.72) | < 0.001 |
| **Citrate** | 0.82 (0.62-1.09) | 0.166 |
| **Alanine** | 0.74 (0.54-1.03) | 0.074 |

*Metabolite concentrations were z-standardized; Hazard Ratios (HR) refer to an increase of one standard deviation. Reference categories: No tumor residual, FIGO I/II, No PARP inhibitor. CI = confidence interval.*

**Table S6C: Interaction analysis of urine metabolites with bevacizumab and PARP inhibitor therapy for overall survival.**

| ***Interactions between*** | **HR (95% CI)** | ***p.value*** |
| --- | --- | --- |
| **Bevacizumab:Glycine** | 0.63 (0.19-2.05) | 0.441 |
| **PARPi:Glycine** | 0.74 (0.14-3.89) | 0.723 |
| **Bevacizumab:Citrate** | 1.16 (0.70-1.90) | 0.567 |
| **PARPi:Citrate** | 1.32 (0.69-2.53) | 0.402 |
| **Bevacizumab:Alanine** | 0.60 (0.26-1.38) | 0.228 |
| **PARPi:Alanine** | 1.02 (0.31-3.39) | 0.979 |

*HR = hazard ratio, CI = confidence interval.*

**Table S6D: Association of the metabolite risk score with overall survival according to maintenance therapy regimens**

| ***Therapy*** | ***N*** | ***Events*** | ***HR (95%CI)*** | ***p.val.adj*** | ***p.interaction*** |
| --- | --- | --- | --- | --- | --- |
| **No bevacizumab** | 94 | 38 | 2.30  (1.43-3.72) | <0.001 |  |
| **Bevacizumab** | 78 | 48 | 4.07  (1.12-14.76) | 0.033 |  |
|  |  |  |  |  | 0.680 |
| **No PARP inhibitor** | 149 | 73 | 2.69  (1.78-4.06) | <0.001 |  |
| **PARP inhibitor** | 23 | 13 | 1.01  (0.07-13.70) | 0.996 |  |
|  |  |  |  |  | 0.407 |

*Hazard ratios (HR) for the metabolite risk score were calculated using multivariable Cox regression, adjusted for residual tumor and FIGO stage. P-values for interaction (p.interaction) represent the significance of the interaction term between the risk score and the respective therapy. CI = confidence interval.*

**Table S7A: Univariate Cox analysis of HGSOC histology for overall survival.**

| ***HR (95% CI)*** | ***p.value*** |
| --- | --- |
| 5.95 (2.17-16.32) | < 0.001 |

*HR = hazard ratio, CI = confidence interval.*

**Table S7B: Multivariable Cox model including clinical variables, HGSOC status, and the three-metabolite signature.**

| ***Variable*** | ***HR (95% CI)*** | ***p.value*** |
| --- | --- | --- |
| Surgical outcome | 3.39 (2.00-5.74) | < 0.001 |
| FIGO | 1.14 (0.49-2.67) | 0.756 |
| HGSOC-Status | 4.45 (1.37-14.42) | 0.013 |
| Glycine | 1.51 (1.21-1.88) | < 0.001 |
| Citrate | 0.76 (0.57-0.999) | 0.049 |
| Alanine | 0.80 (0.62-1.02) | 0.074 |

*HR = hazard ratio, CI = confidence interval.*

**Table S7C: Interaction analysis of individual urine metabolites with HGSOC status for overall survival.**

| ***Interactions between*** | ***HR (95%CI)*** | ***p.value*** |
| --- | --- | --- |
| HGSOC:Glycine | 0.61 (0.35-1.07) | 0.084 |
| HGSOC:Citrate | 2.27 (0.55-9.36) | 0.259 |
| HGSOC:Alanine | 0.38 (0.08-1.78) | 0.217 |

*HR = hazard ratio, CI = confidence interval.*

**Table S7D: Association of the metabolite risk score with overall survival according to histologic subgroup.**

| ***Therapy*** | ***n*** | ***Events*** | ***HR (95%CI)*** | ***p.value*** | ***p.interaction*** |
| --- | --- | --- | --- | --- | --- |
| Other | 26 | 4 | 3.13 (1.23-7.94) | 0.016 |  |
| HGSOC | 127 | 77 | 2.62 (1.16-5.89) | 0.020 | 0.424 |

*HR = hazard ratio, CI = confidence interval.*

**Table S8: Likelihood ratio test for addition of the plasma metabolite signature to Model 3.**

| ***comparison*** | ***Δdf*** | ***Δχ²*** | ***p.value*** |
| --- | --- | --- | --- |
| M3 [surgical outcome + FIGO+ glycine + alanine + citrate]  vs  M3 plus plasma-signature [alanine_high, acetoacetate_low, 3-hydroxybutyrate_low] | 1 | 1.93 | 0.164 |

*All metabolites are z standardized. Δχ² = difference in the chi-square statistic between two nested models (equivalent to 2 × Δ log-likelihood).*
